# *Trichomonas vaginalis*-shaped inflammatory microenvironment drives macrophage immune signaling and modulates cervical cancer cell proliferation and migration

**DOI:** 10.1186/s13071-026-07616-6

**Published:** 2026-08-12

**Authors:** Ching-Yun Huang, Ya-Wen Shih, Chi-Hong Ho, Shu-Fang Chiu, Hsin-Yun Yeh, Lih-Chyang Chen, Hsin-Chung Lin, Hong-Wei Gao, Kuo-Yang Huang

**Affiliations:** 1 Graduate Institute of Medical Sciences, College of Medicine, National Defense Medical University, Taipei City, 114 Taiwan; 2 Host-Parasite Interactions Laboratory, National Defense Medical University, Taipei City, 114 Taiwan; 3 School of Nursing, National Taipei University of Nursing and Health Sciences, Taipei City, 112 Taiwan; 4 Department of Obstetrics and Gynecology, Taipei Veterans General Hospital, Taipei City, 112 Taiwan; 5 Department of Obstetrics and Gynecology, School of Medicine, National Defense Medical University, Taipei City, 114 Taiwan; 6 Department of Obstetrics and Gynecology, School of Medicine, National Yang Ming Chiao Tung University, Taipei City, 112 Taiwan; 7 Department of Inspection, Taipei City Hospital, Renai Branch, Taipei City, 106 Taiwan; 8 Graduate Institute of Pathology and Parasitology, College of Medicine, National Defense Medical University, Taipei City, 114 Taiwan; 9 School of Medicine, College of Medicine, MacKay Medical University, New Taipei City, 252 Taiwan; 10 Department of Optometry, College of Medicine, MacKay Medical University, New Taipei City, 252 Taiwan; 11 Department of Pathology, School of Medicine, College of Medicine, National Defense Medical University, Taipei City, 114 Taiwan; 12 Division of Clinical Pathology, Department of Pathology, Tri-Service General Hospital, Taipei, 114 Taiwan; 13 Department of Pathology, Tungs’ Taichung Metroharbor Hospital, Taichung City, 435 Taiwan

**Keywords:** *Trichomonas vaginalis*, Macrophages, Inflammatory microenvironment, Immune signaling, Cervical cancer

## Abstract

**Background:**

*Trichomonas vaginalis* (TV) infects more than 275 million individuals annually worldwide. However, the effects of the TV-induced inflammatory microenvironment on macrophage immune signaling and cervical cancer cell behavior remain unclear. Here, we investigated macrophage responses to the in vitro TV-induced inflammatory milieu and evaluated its effects, with or without macrophages, on cervical cancer cell proliferation and migration.

**Methods:**

We characterized cytokine profiles and their associated signaling pathways in macrophages after short- and long-term exposure to conditioned medium from TV-stimulated ectocervical cells (TCM). Additionally, the roles of TCM-enriched IL-6, CXCL-1, and IP-10 in regulating TCM-activated pathways were validated. Furthermore, the effects of TCM and the conditioned medium from TCM-stimulated THP-1 macrophages (T-TCM) on HeLa cell proliferation and migration were investigated.

**Results:**

Short-term stimulation of THP-1 macrophages with TCM significantly increased the secretion of SCGF-β and G-CSF, while suppressing IP-10 production. In contrast, prolonged TCM exposure led to elevated levels of G-CSF, IP-10, IL-1ra, and IL-6. Mechanistically, short-term TCM stimulation enhanced activation of the PI3K, STAT3, and NF-κB signaling pathways, promoting the release of SCGF-β, G-CSF, and IP-10. Conversely, long-term TCM stimulation markedly activated the PI3K, STAT1, and p38 MAPK pathways, leading to increased secretion of IP-10, IL-1ra, and IL-6 but decreased G-CSF production. Moreover, TCM treatment enhanced HeLa cell proliferation and migration, whereas T-TCM exerted suppressive effects on these processes.

**Conclusions:**

Collectively, our findings provide novel insights into the immunomodulatory roles of the TV-induced inflammatory microenvironment in shaping macrophage immune signaling cascades and influencing cervical cancer cell behavior.

**Graphical Abstract:**

**Figure:**
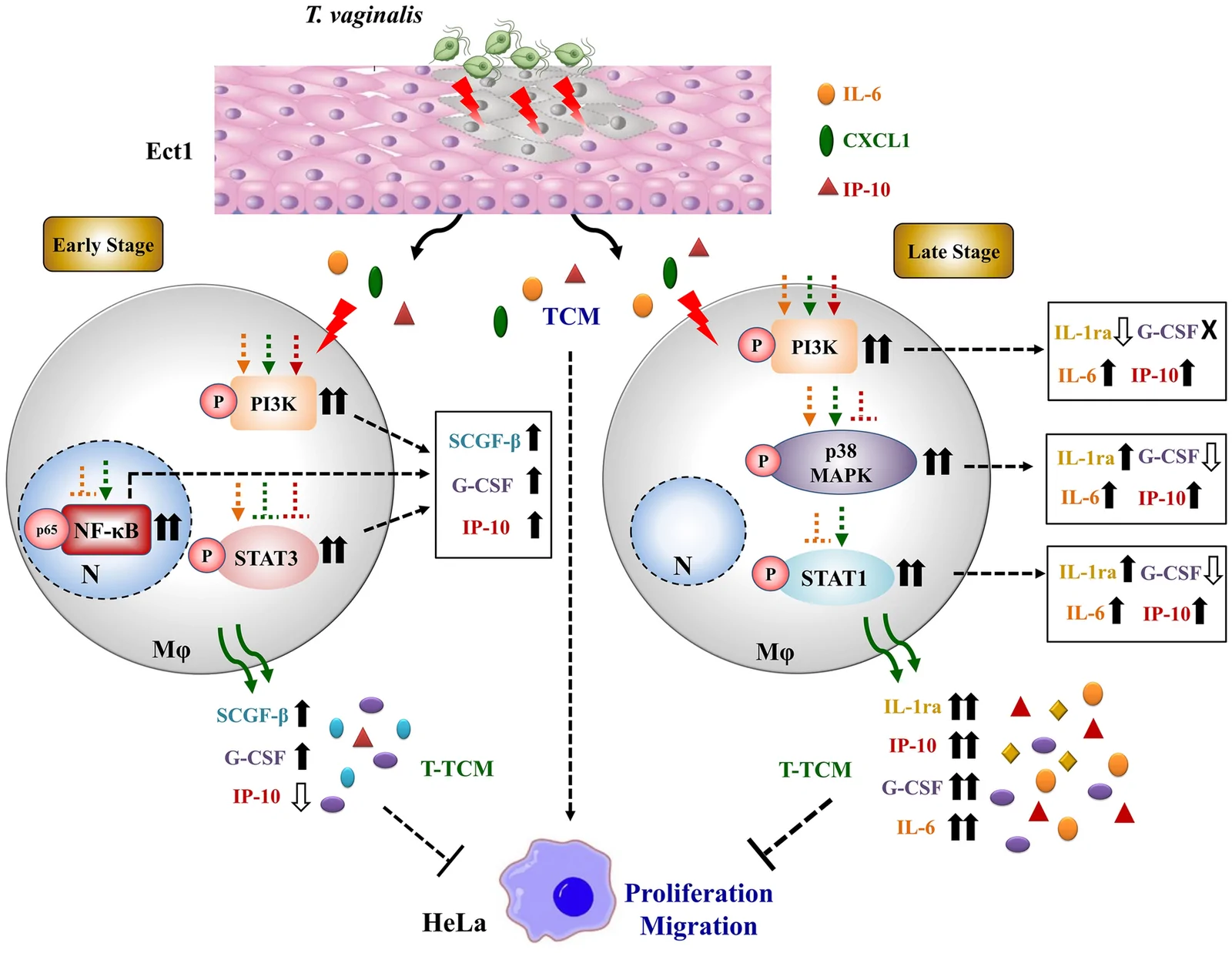

**Supplementary Information:**

The online version contains supplementary material available at https://doi.org/10.1186/s13071-026-07616-6.

## Background

*Trichomonas vaginalis* (TV) is a parasitic protozoan that causes trichomoniasis, the most common non-viral sexually transmitted infections (STIs) worldwide [1]. According to the World Health Organization (WHO), an estimated 156 million new cases of TV infection occurred among individuals aged 15 to 49 years in 2020 [2]. In women, the parasite primarily colonizes the lower urogenital tract, while in men, it is typically found in the prostate and urethra [3]. Clinical manifestations in females include vaginitis, vaginal discharge, pruritus, dysuria, dyspareunia, and the presence of a strawberry cervix––a characteristic punctate hemorrhagic appearance of the vaginal mucosa [4, 5]. In men, TV infection may present as urethritis, prostatitis, or epididymitis [6]. Moreover, trichomoniasis has been associated with an increased risk of cervical and prostate cancers [7, 8], infertility [9], and human immunodeficiency virus (HIV) acquisition [10, 11].

Macrophages are myeloid immune cells distributed throughout body tissues, where they serve as key regulators of immune homeostasis by recognizing pathogens, clearing cellular debris, and orchestrating inflammatory responses to maintain and defend host tissues. These versatile cells contribute to both innate and adaptive immunity [12, 13]. Previous research has demonstrated that TV activates Toll-like receptor-2 (TLR2), a pattern recognition receptor involved in detecting microbial components, thereby stimulating the secretion of pro-inflammatory cytokines, including interleukin-6 (IL-6), interferon-gamma (IFN-γ), and tumor necrosis factor-alpha (TNF-α), in mouse peritoneal macrophages [14]. Additionally, TV has been reported to stimulate macrophages to produce IL-6, TNF-α, and IL-8, further exacerbating local inflammation [15]. Despite its pro-inflammatory effects, TV also employs immune evasion strategies in the hosts, including the inhibition of nuclear factor kappa-light-chain-enhancer of activated B cells (NF-κB) signaling and the induction of macrophage apoptosis [16]. Recent studies have demonstrated that TV and its extracellular vesicles (TV-EVs) can induce NLRP3 inflammasome activation in human macrophages, leading to caspase-1 activation and the subsequent secretion of IL-1β [17, 18]. The pro-inflammatory mediators induced by TV may serve as key regulators of immune signaling and facilitate the activation and recruitment of additional immune cells to the site of infection. These findings highlight the complex interplay between TV infection and macrophage-mediated immune responses, potentially modulating the development of TV-associated STIs and cancers [19].

TV can adhere to and lyse host epithelial cells, leading to extensive tissue damage and disruption of the epithelial barrier [20]. This process creates a pro-inflammatory microenvironment characterized by the release of various cytokines. Previous studies have demonstrated that the inflammatory milieu induced by TV infection can promote the proliferation and migration, as well as enhance the secretion of CXCL1, CCL2, and IL-6 in prostate cancer cells [21]. Additionally, TV infection could upregulate TLR2, 4, and 9 gene expression via the p38 mitogen-activated protein kinase (MAPK) signaling pathway in HeLa cells, modulating p38 MAPK-dependent IL-8 and TNF-α secretion [22]. Persistent infection with TV has been associated with chronic inflammation, epithelial exfoliation, and disruption of local immune defenses, all of which contribute to cervical cancer development [23, 24]. Epidemiological data indicate that women infected with TV have a higher risk of developing cervical cancer compared to uninfected individuals [23]. It is noteworthy that cytokines play vital roles in modulating cervical cancer progression. Certain cytokines, such as IL-2, IFN-γ, IL-12, IL-21, IL-10, IL-32, IL-37, and transforming growth factor-β (TGF-β1), are considered to exert tumor-suppressive effects. Conversely, others including IL-1, IL-6, IL-8, IL-10, IL-17, IL-32, TGF-β1, MCP-1, and RANTES may promote tumor development. Notably, IL-10, IL-32, and TGF-β1 exhibit dual functions, acting as both tumor suppressors and promoters under different conditions [25, 26]. It remained to be determined whether TV-induced cytokine secretion from the host cells directly contributes to cervical cancer development or to other TV-associated STIs.

The molecular mechanisms governing macrophage responses within the TV-induced microenvironment, as well as the potential influence of this microenvironment on cervical cancer cell progression, remain poorly understood. In this study, we characterized the cytokine profiles and associated immunomodulatory signaling pathways in macrophages exposed to an in vitro TV-induced microenvironment. The inflammatory microenvironment was generated through the interaction between TV and ectocervical cells (Ect1). Our findings demonstrate for the first time that the conditioned medium of Ect1 cells stimulated with TV (TCM) enhances the activation of multiple signaling pathways in THP-1 macrophages, including the PI3K, signal transducer and activator of transcription 1 (STAT1), STAT3, NF-κB, and p38 MAPK, thereby modulating cytokine production. We previously demonstrated that IL-6, CXCL1, and IP-10 are highly secreted in Ect1 cells infected with TV [27]. Hence, we further elucidated the roles of these TCM-containing cytokines in altering immune signaling cascades in macrophages. Moreover, we assessed the effects of both TCM and the conditioned medium from THP-1 macrophages stimulated with TCM (T-TCM) on the behavior of cervical cancer cells. Our results revealed that the TCM promotes proliferation and migration of HeLa cells, whereas T-TCM exerts a suppressive effect on these processes. These findings highlight the dynamic interplay between epithelial cells, macrophages, and cancer cells in the in vitro TV-induced inflammatory microenvironment.

## Methods

### Parasites and cell culture

The TV strain (ATCC 30236) was maintained in YI-S medium supplemented with 10% horse serum and 1% glucose at 37 °C. The human monocytic cell line THP-1 (ATCC TIB-202) was grown in RPMI 1640 medium containing 10% fetal bovine serum (FBS) and incubated in 5% CO_2_ at 37 °C. The human ectocervical epithelial cell line Ect1/E6E7 (ATCC CRL-2614^™^) was cultured in keratinocyte serum-free medium (KSFM) (Gibco, Thermo Fisher Scientific, Inc., Waltham, MA, USA) supplemented with 0.1 ng/ml recombinant human epidermal growth factor (EGF), 0.05 mg/ml bovine pituitary extract, and 0.4 mM calcium chloride, and incubated in 5% CO₂ at 37 °C. HeLa cells were maintained in MEM-α supplemented with 10% FBS, 1% penicillin–streptomycin, and 1% sodium pyruvate at 37 °C in a humidified 5% CO₂ incubator.

### Collection of conditioned medium from TV-stimulated Ect1 cells and TCM-stimulated THP-1 macrophages

Ect1 cells were seeded at a density of 2 × 10^6^ cells per 6-cm dish and incubated overnight in 5% CO_2_ at 37 °C. The next day, cells were washed three times with PBS, and 2 ml of KSFM was added. Cells were then incubated with or without TV (2 × 10⁶ cells) for 10 h. After incubation, the parasites were removed by centrifugation at 3000 rpm for 10 min, and the culture supernatants were collected. The supernatants derived from Ect1 cells stimulated with or without TV were defined as TV-conditioned medium (TCM) and control conditioned medium (CM), respectively. THP-1 cells were seeded a density of 5 × 10^6^ cells per 6-cm dish and differentiated into macrophage-like cells by treatment with 50 ng/mL phorbol 12-myristate 13-acetate (PMA; Sigma-Aldrich, USA) for 24 h at 37 °C in 5% CO₂. Following differentiation, the culture medium was removed, and cells were washed three times with PBS. The cells were then incubated with TCM for either 4 h or 24 h, and the resulting culture supernatants were collected and designated as 4 h T-TCM and 24 h T-TCM, respectively.

### Multiplex immunoassay and enzyme-linked immunosorbent assay (ELISA)

THP-1 macrophages were seeded at a density of 2 × 10^5^ cells per well in 24-well plates and treated with 50 ng/ml PMA for 24 h at 37 °C in a 5% CO₂ incubator. The culture medium was removed, and cells were washed with PBS, followed by stimulation with 250 µl of either TCM or CM for different time intervals, including short-term (4 and 8 h) and long-term (24, 48, and 72 h) treatments. Following incubation, supernatants were collected and analyzed for 48 human cytokines using a Bio-Plex multiplex immunoassay system (Bio-Rad, USA). Conditioned medium from Ect1 cells stimulated with or without TV for 10 h served as controls. To validate the cytokine profiles obtained from the multiplex immunoassay, selected cytokines were further quantified using ELISA kits (R&D Systems, USA). Supernatants collected from THP-1 cells after short- and long-term stimulation with TCM or CM were subjected to ELISA following the manufacturer’s protocols.

### Western blot analysis

Cells were harvested and lysed in RIPA protein extraction solution (Bio Future, Taiwan). Protein concentrations were measured with a BCA protein assay kit (Bio Future, Taiwan). Proteins were separated by 12% sodium dodecyl sulfate polyacrylamide gel electrophoresis (SDS-PAGE) and transferred to polyvinylidene fluoride (PVDF) membranes (Bio-Rad, USA). Membranes were blocked with 5% bovine serum albumin in Tris-buffered saline with 0.1% Tween-20 (TBST) for 1 h at room temperature (RT), and then incubated overnight at 4 °C with primary antibodies targeting the following proteins: phosphor-phosphoinositide 3-kinase (PI3K), phosphor-AKT, phosphor-STAT1, phosphor-STAT3, phosphor-NF-κB, phosphor-extracellular signal-regulated kinase (ERK), phosphor-p38 MAPK, E-cadherin, Vimentin, Slug, and β-actin (1:1000, Cell Signaling Technology Inc., MA, USA). After washing with TBST, membranes were incubated with Goat anti-rabbit HRP-conjugated IgG (1:5000, Croyez Bioscience, Taiwan) for 1 h at RT. Protein bands were detected with Immobilon Western Chemiluminescent HRP Substrate (Merck Millipore, USA) and signals were measured with SPOT Xplorer (SPOT Imaging, USA). Quantification of protein bands was performed using Image J.

### Neutralizing antibody treatment

To examine the regulatory roles of IL-6, CXCL-1, and IP-10 in TCM-enhanced signaling pathways in THP-1 macrophages at both early and late stages, TCM was pre-incubated with different concentrations of neutralizing antibodies (1, 2, and 5 µg/ml) against IL-6 (R&D Systems, USA), CXCL1(Sigma-Aldrich, USA), and IP-10 (R&D Systems, USA) at 37 °C for 1 h. The neutralizing efficiency of antibodies against their targets was validated using ELISA. The antibody-treated TCM was then incubated with THP-1 macrophages for either 4 h or 24 h. Following stimulation, the activation of TCM-enhanced pathways was assessed in THP-1 macrophages by Western blot analysis as mentioned above.

### Immunofluorescence assay

THP-1 cells were seeded at a density of 4 × 10^5^ cells per coverslip and treated with 50 ng/ml PMA for 24 h in 5% CO_2_ at 37 °C. The cells were washed and co-cultured with TCM for 2 h. After incubation, the TCM was removed, and the cells were washed, fixed with 4% paraformaldehyde for 20 min at RT, and then blocked with 0.3% bovine serum albumin for 1 h. The coverslips were then incubated with anti-NF-κB (1:200; Cell Signaling, USA) overnight at 4 °C. Cells were washed and stained with Alexa Fluor 488 dye (1:500; Invitrogen, USA) at RT for 1 h. The nuclei were stained with 4',6-diamidino-2-phenylindole (DAPI, Sigma-Aldrich; USA). Fluorescence was measured using a confocal microscope (LSM 980, Zeiss, Germany).

### Cell viability assay

Cell viability was evaluated using the CCK-8 Kit (Sigma-Aldrich, USA) according to the manufacturer’s instructions. THP-1 macrophages or HeLa cells were seeded in 24-well plates at a density of 2 × 10^5^ cells/well and incubated overnight at 37 °C in 5% CO₂. To assess the effect of TCM on macrophage viability, THP-1 cells were stimulated with either TCM or CM for short-term (4 and 8 h) and long-term (24, 48, and 72 h) durations. To examine the role of macrophages in HeLa cell proliferation in a TV-induced microenvironment, HeLa cells were co-cultured with CM, TCM, 4 h T-TCM, or 24 h T-TCM. Subsequently, 20 μl of the CCK-8 reagent was added to each well, and absorbance was measured at 450 nm using a microplate reader after incubation for 30 min at 37 °C.

### Transwell migration assay

HeLa cells were seeded at a density of 2 × 10^5^ cells and placed on the upper layer of a cell culture insert with a permeable membrane (Costar, USA), while the TCM, 4 h T-TCM, or 24 h T-TCM agent was placed below the membrane. After incubation for 24 h, cell samples were harvested, and the cells adhering to the lower surface of the chamber were fixed with 4% paraformaldehyde for 30 min. The samples were then rinsed three times with PBS, stained with 0.5 mM crystal violet dye for 30 min, and subsequently observed and photographed under a microscope (Olympus CKX41 microscope, Japan).

### Statistical analysis

Quantitative data are presented as the mean ± standard error of the mean (SEM) from at least three independent biological replicates (n ≥ 3), with each biological replicate analyzed in technical triplicate. Statistical differences between two experimental groups were evaluated using a two-tailed, unpaired Student’s t-test. For multiplex immunoassay screenings (n = 1), the data were used solely for candidate selection and were subsequently validated by ELISA. All statistical analyses and graphical presentations were performed using GraphPad Prism software. A *p* value < 0.05 was considered statistically significant.

## Results

### Short and long-term stimulation with TV-induced condition medium enhances various cytokine secretions in THP-1 macrophages

**Fig. 1:**
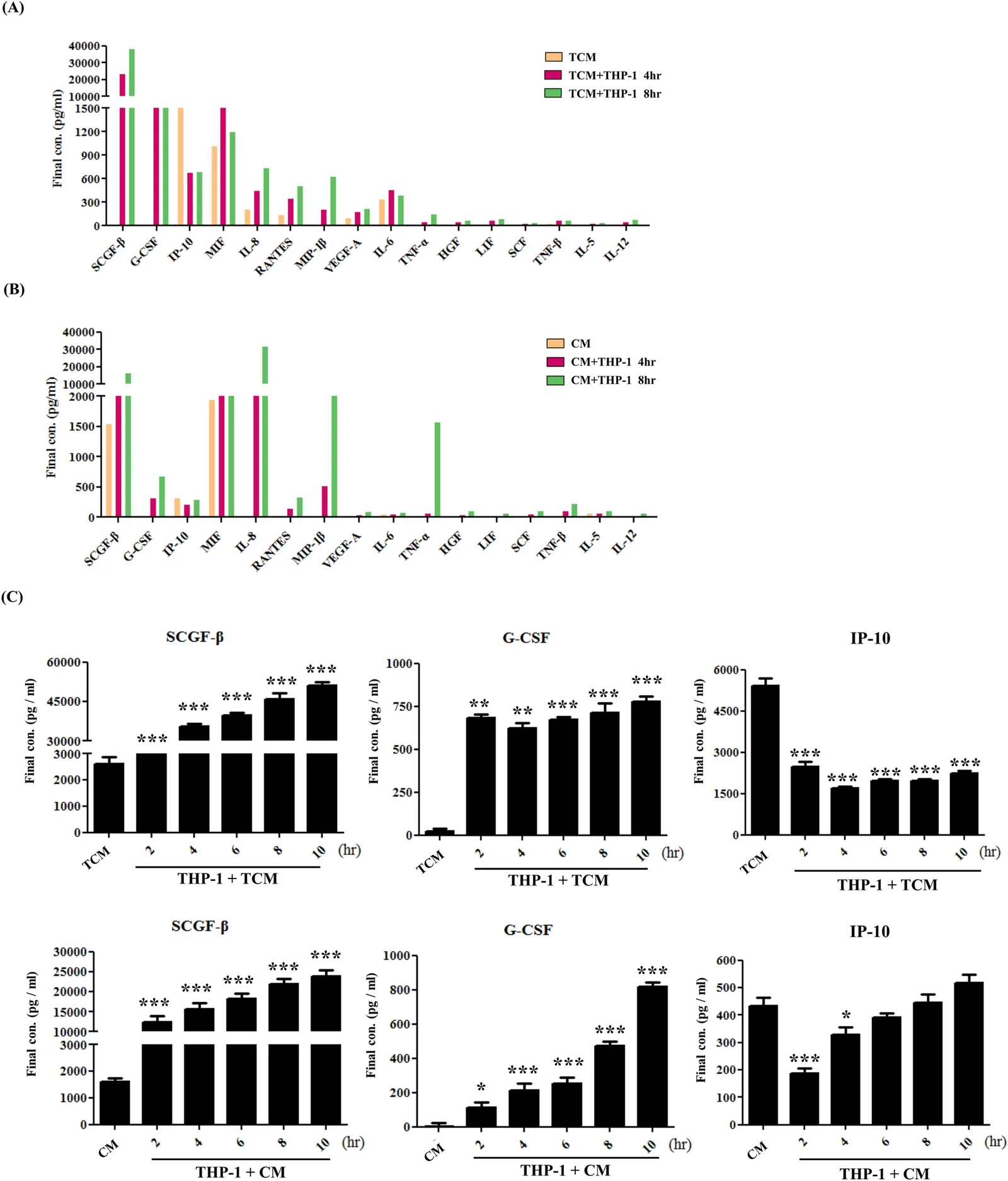
Short-term exposure to TCM markedly enhances the secretion of SCGF-β and G-CSF, while suppressing IP-10 production in THP-1 macrophages. THP-1 macrophages (2 × 10^5^ cells) were co-cultured with **A** TCM or **B** CM for 4 and 8 h. The co-culture supernatants were collected and analyzed using a multiplex immunoassay to screen 48 common human cytokines. Culture supernatants from Ect1 cells cultured alone (CM) or co-cultured with TV (TCM) for 10 h served as controls. **C** Culture supernatants from THP-1 macrophages treated with TCM or CM for 2, 4, 6, 8, and 10 h were analyzed for SCGF-β, G-CSF, and IP-10 levels using ELISA. *p < 0.05, **p < 0.01, and ***p < 0.001

We previously demonstrated that TV infection induces the secretion of IL-6, IP-10, and CXCL1 in Ect1 cells, with peak levels observed at 10 h post-infection [27]. Hence, we collected the conditioned medium from a 10 h TV-Ect1 co-culture (TCM) to represent the severe inflammatory microenvironment generated during TV infection. The condition medium obtained from Ect1 cultures without TV exposure (CM) served as the uninfected condition. Macrophages play a predominant role in inflammation, with their phenotypes and functions regulated by the surrounding microenvironment [28]. We first evaluated the viability of THP-1 macrophages after prolonged stimulation with either CM or TCM for 24, 48, and 72 h. Notably, both CM- and TCM-treated THP-1 macrophages exhibited significantly increased viability after 72 h compared to the untreated control (Fig. S1). To investigate the impact of the TV-induced inflammatory microenvironment on macrophage cytokine secretion, we assessed cytokine profiles in THP-1 macrophages after short- and long-term stimulation with TCM compared to CM. To distinguish whether the observed cytokine profiles resulted from residual mediators present in the TCM or from de novo cytokine secretion by macrophages, cell-free TCM and CM were included as parallel baseline controls. Upon short-term stimulation with TCM for 4 and 8 h, multiplex immunoassay revealed significantly elevated secretion of stem cell growth factor-β (SCGF-β) and granulocyte colony-stimulating factor (G-CSF), while IP-10 secretion was inhibited in THP-1 macrophages compared to the TCM alone group (Fig. 1A). Although a similar trend was observed in CM-stimulated THP-1 macrophages (Fig. 1B), the overall magnitude of cytokine induction was substantially lower than that elicited by TCM treatment. To validate this differential induction, we performed ELISA to quantify SCGF-β, G-CSF, and IP-10 secretion in THP-1 macrophages treated with TCM or CM over various time points (2, 4, 6, 8, and 10 h) relative to their cell-free medium baselines (Fig. 1C). Consistent with the multiplex immunoassay data, TCM stimulation significantly increased SCGF-β and G-CSF levels from 2 to 10 h, whereas IP-10 secretion was notably reduced as early as 2 h post-stimulation. Together, these in vitro findings indicate that short-term exposure to the TV-induced inflammatory microenvironment enhances the secretion of SCGF-β and G-CSF, while suppressing IP-10 production in THP-1 macrophages.

**Fig. 2:**
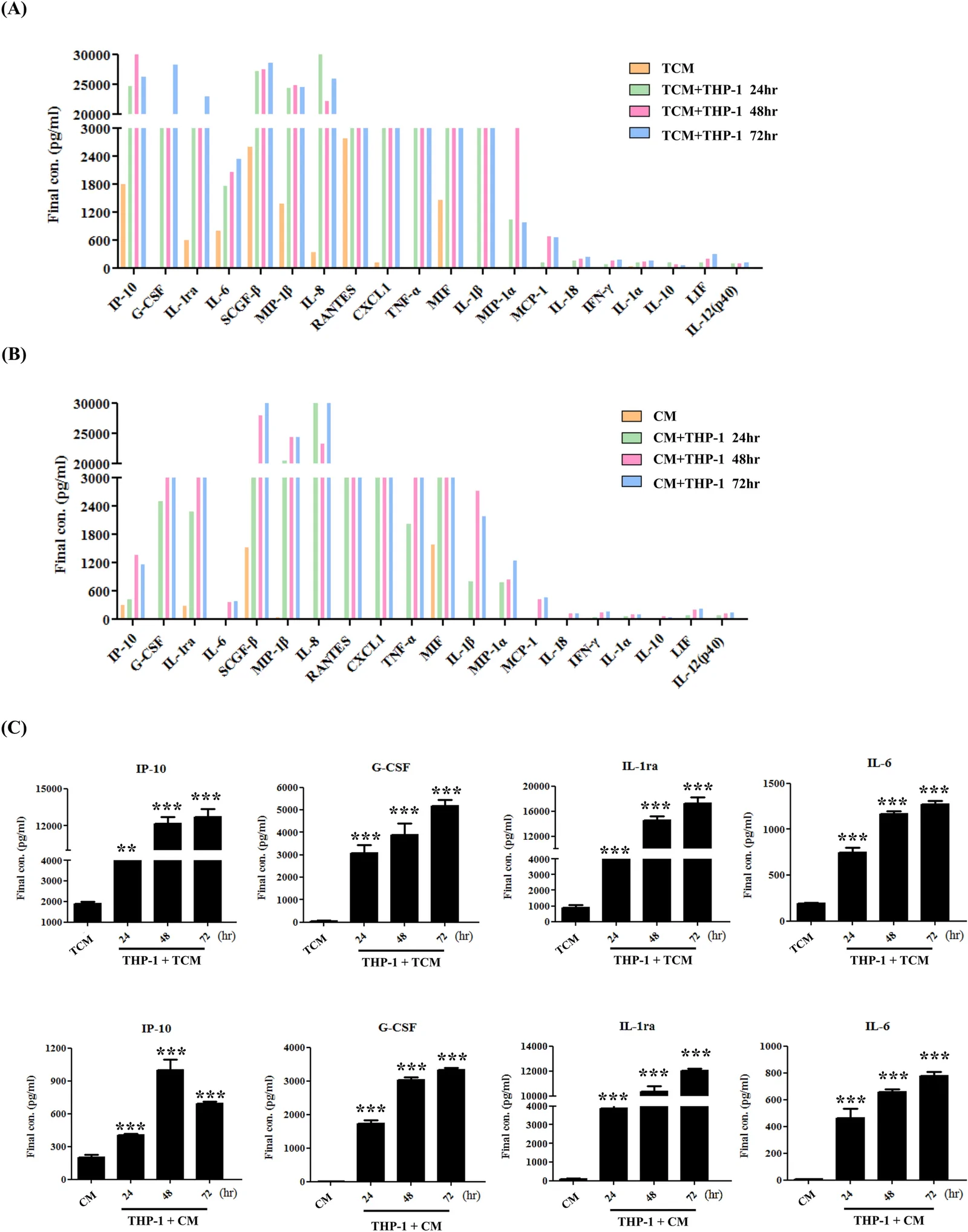
Long-term exposure to TCM significantly enhances the secretion of IP-10, G-CSF, IL-1ra, and IL-6 in THP-1 macrophages. THP-1 macrophages (2 × 10^5^ cells) were co-cultured with **A** TCM or **B** CM for 24, 48, and 72 h. Supernatants were collected and analyzed using a multiplex immunoassay to screen 48 common human cytokines. Culture supernatant collected from Ect1 cells cultured alone (CM) or co-cultured with TV (TCM) for 10 h served as controls. **C** Supernatants from THP-1 macrophages treated with TCM for 24, 48, and 72 h were analyzed for IP-10, G-CSF, IL-1ra, and IL-6 secretion using ELISA. **p < 0.01 and ***p < 0.001

We further evaluated the secretion of various cytokines released in THP-1 macrophages after long-term stimulation with TCM compared to CM. Multiplex immunoassay revealed that IP-10, G-CSF, IL-1ra, and IL-6 were markedly secreted in THP-1 macrophages after stimulation with TCM for 24, 48, and 72 h relative to the TCM alone group (Fig. 2A, B). Importantly, the overall magnitude of these secretory responses was substantially more robust in TCM-stimulated THP-1 macrophages than in those stimulated with CM, when evaluated against their cell-free baselines. In contrast to the reduced secretion of IP-10 in THP-1 macrophages after short-term stimulation with TCM for 8 h, the release of IP-10 was further enhanced after long-term stimulation for 24 h and retained until at least 72 h. These results were further validated by ELISA (Fig. 2C), which confirmed the cytokine secretion trends observed in the multiplex immunoassay. Together, these findings demonstrate that prolonged exposure to the TV-induced inflammatory microenvironment enhances the secretion of IP-10, G-CSF, IL-1ra, and IL-6 in THP-1 macrophages.

### Short-term stimulation with TCM enhances the activation of PI3K, STAT3, and NF-κB pathways to mediate the secretion of SCGF-β, G-CSF, and IP-10 in THP-1 macrophages

**Fig. 3:**
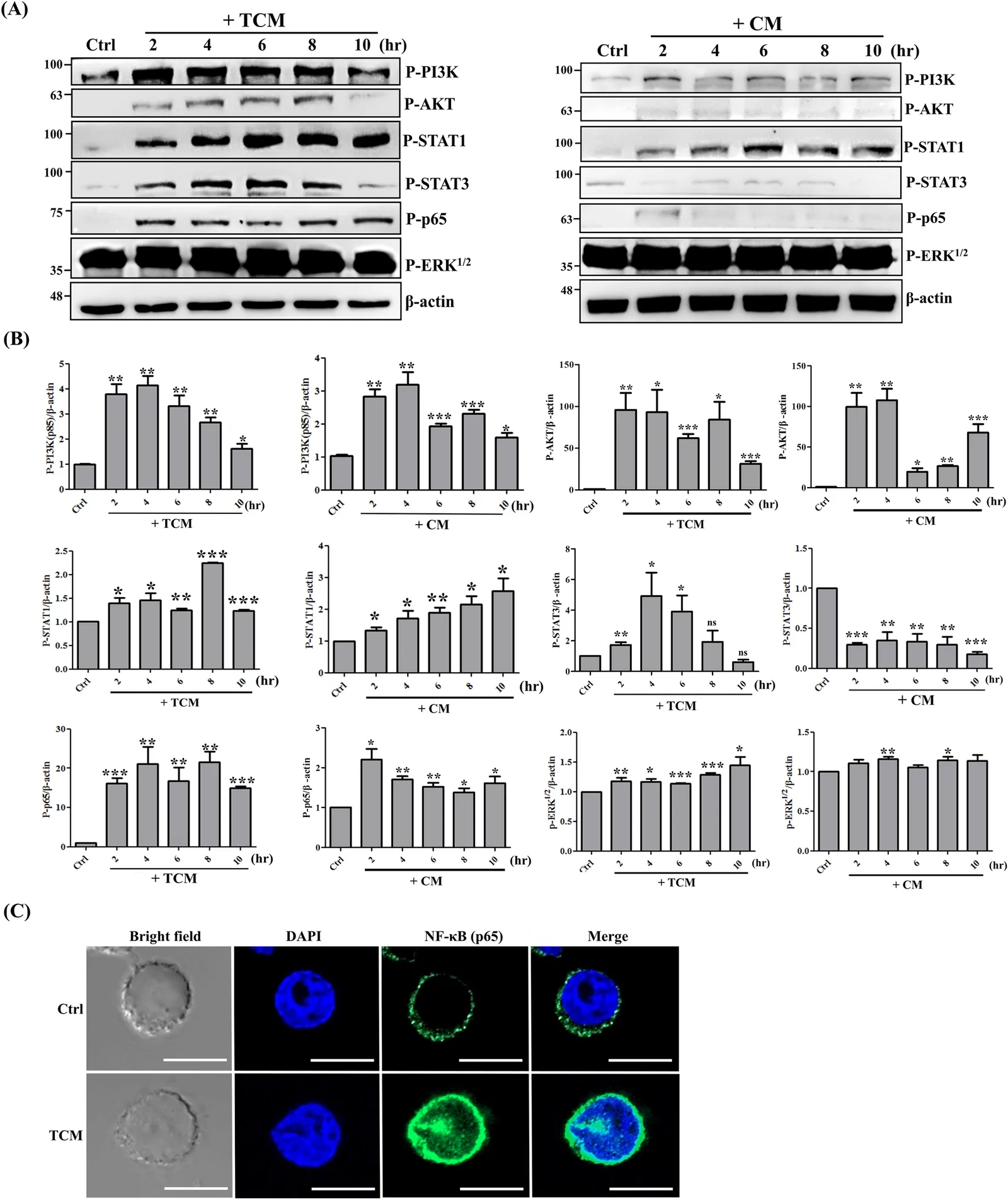
Short-term stimulation with TCM enhances the activation of PI3K, STAT3, and NF-κB p65 pathways in THP-1 macrophages. **A** THP-1 macrophages (5 × 10^6^ cells) were treated with TCM or CM for 2, 4, 6, 8, and 10 h compared with the untreated control (Ctrl). Whole cell lysates were collected and subjected to Western blot analysis to assess the phosphorylation levels of PI3K, AKT, STAT1, STAT3, ERK, and NF-κB P-p65 using specific antibodies. β-actin was used as an internal control for Western blot analysis. **B** Quantification of protein band intensities was performed using ImageJ software. **C** Immunofluorescence analysis was conducted to examine the subcellular localization of NF-κB p65 in THP-1 macrophages treated with TCM for 2 h compared with the untreated control (Ctrl). Cells were stained with an anti-NF-κB p65 antibody (green), and nuclei were counterstained with DAPI (blue). Images were captured using a confocal microscope. Scale bar: 10 µm. *p < 0.05, **p < 0.01, and ***p < 0.001

We next explored the intracellular signaling pathways involved mediating cytokine production in THP-1 macrophages upon stimulation with TV-induced microenvironment. Previous studies have shown that IP-10 plays pivotal roles in human cancer, contributing to chemotaxis, apoptosis, cell growth, angiostasis, and tumor progression. The major involved signaling pathways include PI3K, AKT, STAT1, NF-κB, p38 MAPK, and ERK [29–31]. Additionally, M2 macrophage‐derived G-CSF has been shown to promote epithelial–mesenchymal transition (EMT), migration, and invasion of trophoblasts via the PI3K, AKT, and ERK pathways [32]. Based on these findings, we examined whether the PI3K, AKT, STAT1, STAT3, and NF-κB pathways are activated in THP-1 macrophages following stimulation with TCM or CM over various time intervals. Western blot analysis revealed that phosphorylation of PI3K, AKT, STAT1, NF-κB p65, and ERK were significantly increased in THP-1 macrophages stimulated with either TCM or CM for 2 h relative to the untreated control (Fig. 3A). Specifically, the activation of PI3K, STAT3, and NF-κB p65 were more pronounced in THP-1 macrophages stimulated with TCM compared to those treated with CM (Fig. 3B). Immunofluorescence staining was conducted to verify the activation of NF-κB p65, revealing its nuclear translocation in THP-1 macrophages following 2 h of TCM stimulation (Fig. 3C). These findings indicate that short-term exposure to the in vitro TV-induced inflammatory milieu robustly activates the PI3K, STAT3, and NF-κB signaling pathways in THP-1 macrophages.

**Fig. 4:**
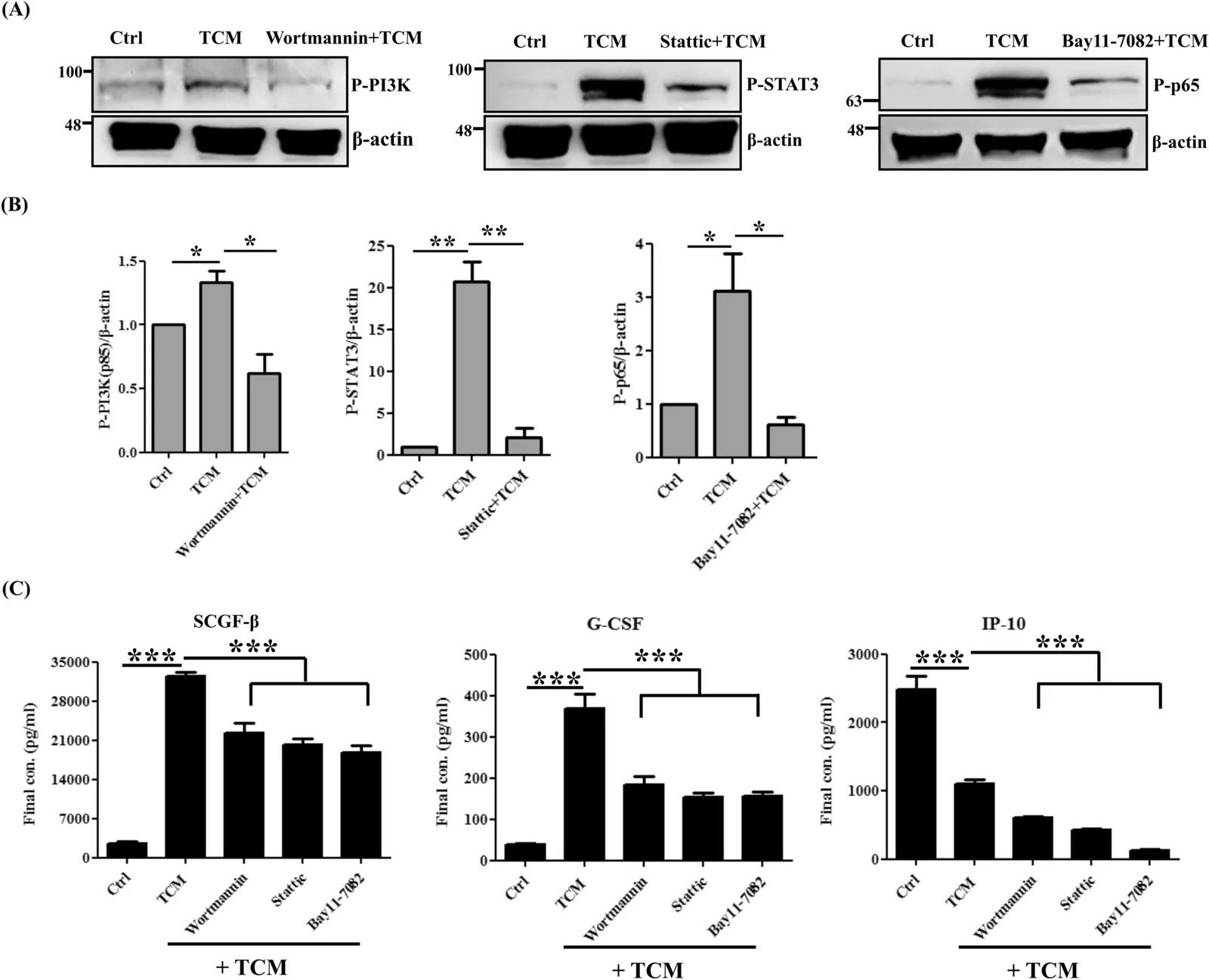
The secretion of SCGF-β, G-CSF, and IP-10 is regulated by the PI3K, STAT3, and NF-κB signaling pathways in THP-1 macrophages upon short-term exposure to TCM. **A** THP-1 macrophages (5 × 10^6^ cells) were pretreated with pathway-specific inhibitors, including Wortmannin (PI3K), Stattic (STAT3), and Bay11-7082 (NF-κB p65), followed by stimulation with TCM for 4 h. Phosphorylation levels of PI3K, STAT3, and NF-κB p65 were analyzed by Western blot analysis and compared with the TCM-treated and untreated control (Ctrl) groups. β-actin served as an internal control. **B** Quantification of protein band intensities was performed using ImageJ software. **C** Culture supernatants from THP-1 macrophages treated with different conditions as mentioned in **(A)** were collected, and the levels of SCGF-β, G-CSF, and IP-10 were measured using ELISA. *p < 0.05, **p < 0.01, and***p < 0.001

To further examine whether TCM-enhanced activation of the PI3K, STAT3, and NF-κB signaling pathways regulates the secretion of SCGF-β, G-CSF, and IP-10 in macrophages, THP-1 macrophages were pretreated with specific inhibitors targeting PI3K (Wortmannin), STAT3 (Stattic), and NF-κB (Bay 11–7082) signaling, followed by stimulation with TCM for 4 h. The effects of these pathway inhibitors were confirmed by Western blot analysis, showing a significant reduction in the phosphorylation of PI3K, STAT3, and NF-κB p65 compared to the TCM-treated group (Fig. 4A, B). Subsequently, we assessed the secretion of TCM-regulated SCGF-β, G-CSF, and IP-10 in THP-1 macrophages following pathway inhibition. It is obviously that THP-1 macrophages pretreated with Wortmannin, Stattic, and Bay 11–7082 significantly suppressed the TCM-induced release of all three cytokines compared to TCM treatment alone for 4 h (Fig. 4C). Collectively, these findings indicate that short-term exposure to the in vitro TV-induced inflammatory milieu strongly activates the PI3K, STAT3, and NF-κB pathways to mediate the secretion of SCGF-β, G-CSF, and IP-10 in THP-1 macrophages.

### Long-term stimulation with TCM enhances the activation of PI3K, STAT1, and p38 MAPK signaling pathways to modulate the secretion of IP-10, G-CSF, IL-1ra, and IL-6 in THP-1 macrophages

**Fig. 5:**
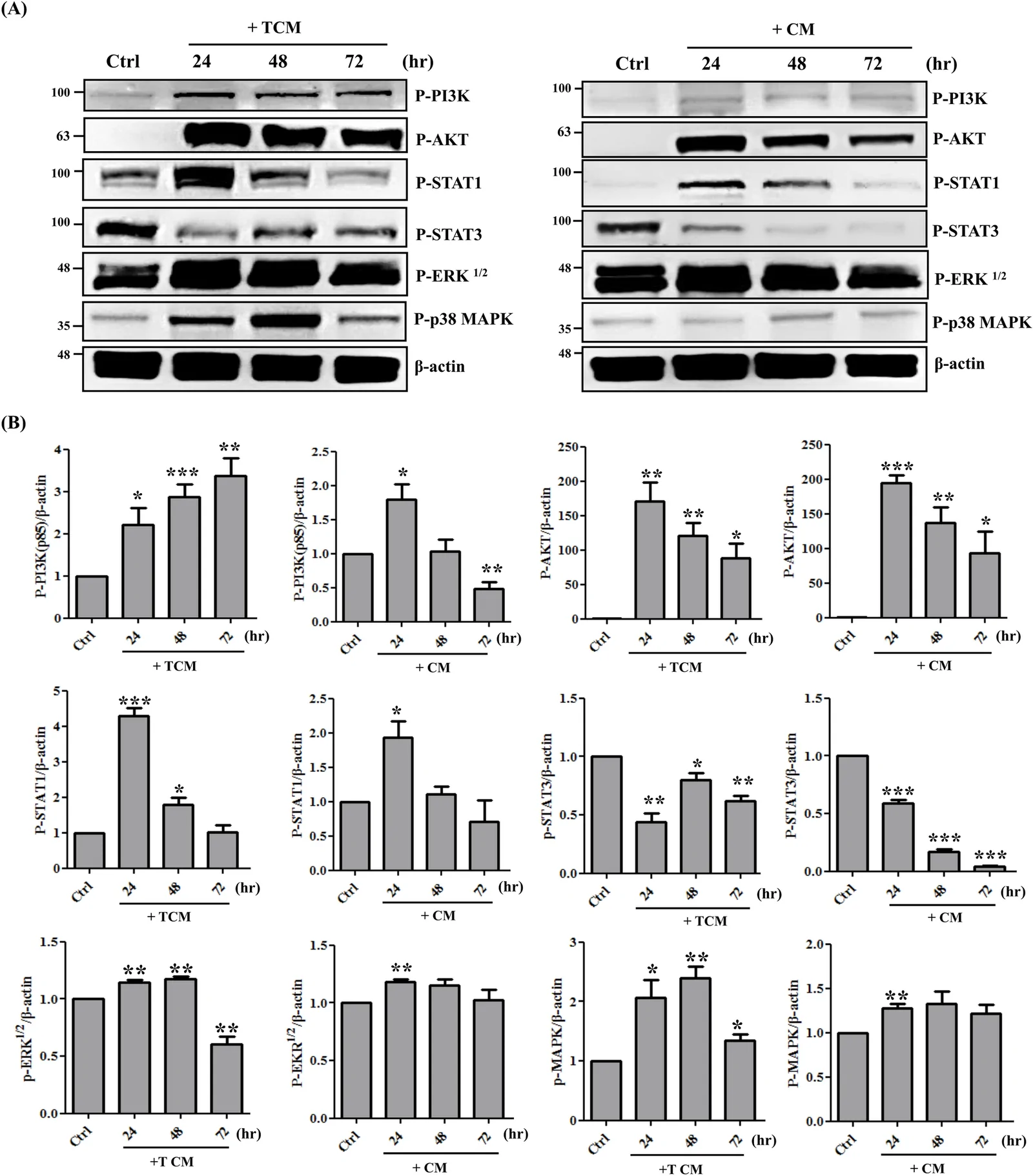
Long-term exposure to TCM enhances the activation of PI3K, STAT1, and p38 MAPK signaling in THP-1 macrophages. **A** THP-1 macrophages (5 × 10^6^ cells) were treated with TCM or CM for 24, 48, and 72 h compared with the untreated control (Ctrl). Whole cell lysates were collected and subjected to Western blot analysis to detect the phosphorylation levels of PI3K, AKT, STAT1, STAT3, ERK, and p38 MAPK using specific antibodies. β-actin served as an internal control. **B** Quantification of protein bands intensities was performed using ImageJ software. *p < 0.05, **p < 0.01, and ***p < 0.001

We next assessed the effects of prolonged stimulation with the TV-induced microenvironment on the activation of intracellular signaling pathways in THP-1 macrophages. Compared to the untreated control, the phosphorylation of PI3K, AKT, STAT1, ERK, and p38 MAPK were significantly elevated in THP-1 macrophages stimulated with TCM or CM for 24 h, whereas the phosphorylation of STAT3 was notably reduced (Fig. 5A, B). Specifically, the activation of PI3K, STAT1, and p38 MAPK was more pronounced in TCM-treated macrophages than in those stimulated with CM. Hence, these findings indicate that long-term exposure to the in vitro TV-induced inflammatory milieu robustly activates specific signaling pathways, such as PI3K, STAT1, and p38 MAPK, in THP-1 macrophages.

**Fig. 6:**
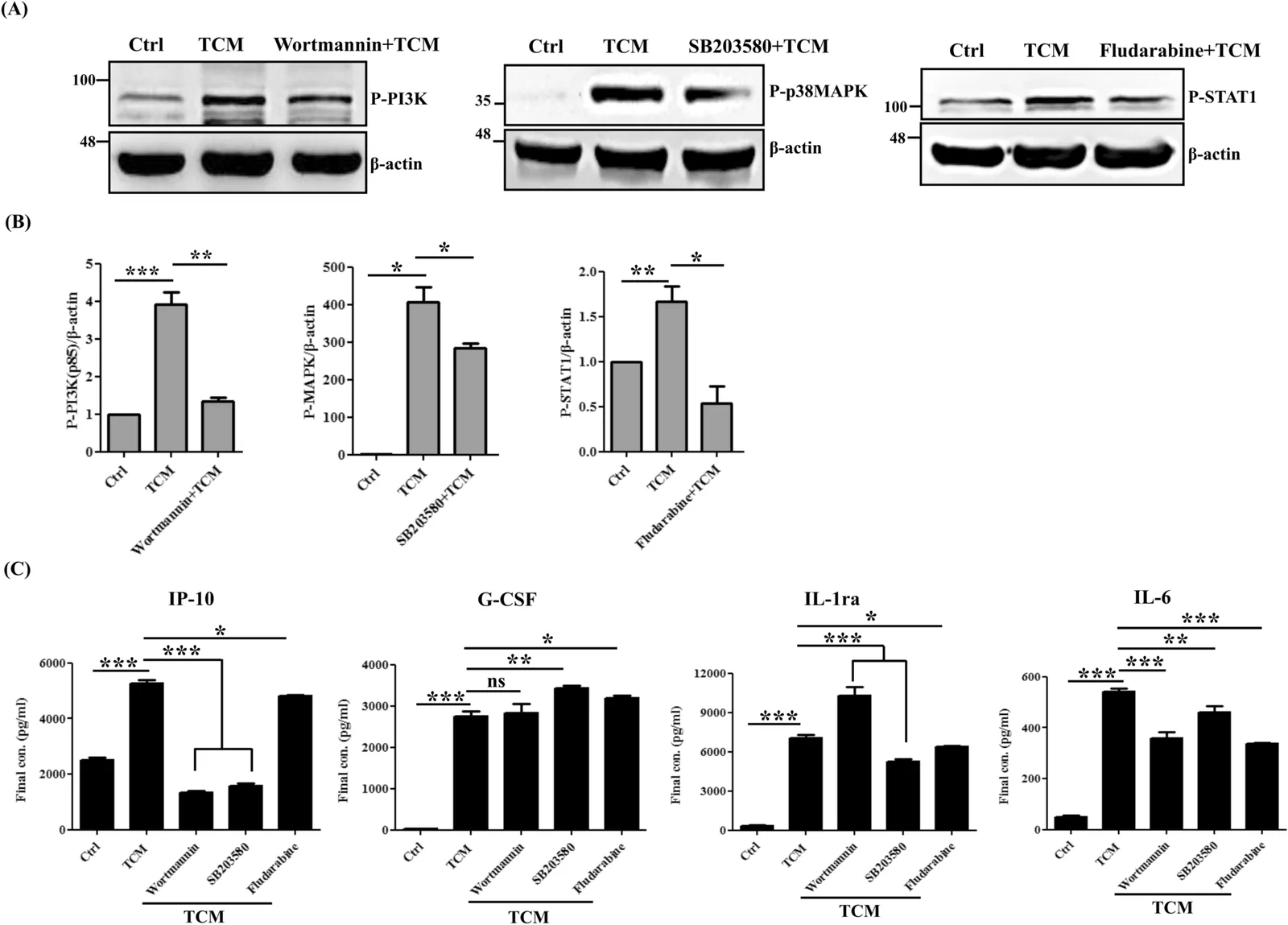
Differential regulation of IP-10, G-CSF, IL-1ra, and IL-6 secretion by PI3K, p38 MAPK, and STAT1 signaling pathways in THP-1 macrophages following long-term exposure to TCM. **A** THP-1 macrophages (5 × 10^6^ cells) were pretreated with pathway-specific inhibitors targeting PI3K (Wortmannin), p38 MAPK (SB203580), or STAT1 (Fludarabine), followed by stimulation with TCM for 24 h. Whole cell lysates were collected, and the phosphorylation levels of PI3K, p38 MAPK, and STAT1 were analyzed by Western blot analysis using specific antibodies compared with TCM-treated and untreated control (Ctrl) groups. β-actin served as a loading control. **B** Quantification of protein band intensities was performed using ImageJ software. **C** Cytokine levels of IP-10, G-CSF, IL-1ra, and IL-6 in THP-1 macrophages under different conditions as mentioned in (**A**) were determined by ELISA. *p < 0.05, **p < 0.01, ***p < 0.001. *ns* Not significant

To determine whether the PI3K, STAT1, p38 MAPK pathways are involved in regulating the secretion of IP-10, G-CSF, IL-1ra, and IL-6 in THP-1 macrophages upon long-term stimulation with TCM for 24 h, cells were pretreated with specific inhibitors targeting PI3K (Wortmannin), p38 MAPK (SB203580), and STAT1 (Fludarabine), followed by measuring cytokine secretion. The phosphorylation of PI3K, p38 MAPK, and STAT1 was markedly suppressed in THP-1 macrophages pretreated with these inhibitors compared to those treated with TCM alone (Fig. 6A, B). Additionally, the secretion of IP-10 and IL-6 was significantly inhibited in THP-1 macrophages pretreated with Wortmannin, while IL-1ra secretion was increased. No significant change in G-CSF secretion was observed after blocking PI3K signaling (Fig. 6C). Furthermore, the secretion of IP-10, IL-1ra, and IL-6 was significantly inhibited in THP-1 macrophages pretreated with the p38 MAPK inhibitor SB203580 and the STAT1 inhibitor Fludarabine, while G-CSF secretion was significantly increased. Collectively, these findings suggest that long-term exposure to TCM markedly activates the PI3K, p38 MAPK, and STAT1 pathways in THP-1 macrophages, which positively regulates the secretion of IP-10 and IL-6, while exerting differential regulatory effects on IL-1ra and G-CSF production.

### TCM-containing IL-6, CXCL1, and IP-10 exert distinct regulatory functions on PI3K, STAT1, STAT3, NF-κB, and p38 MAPK in THP-1 macrophages

**Fig. 7:**
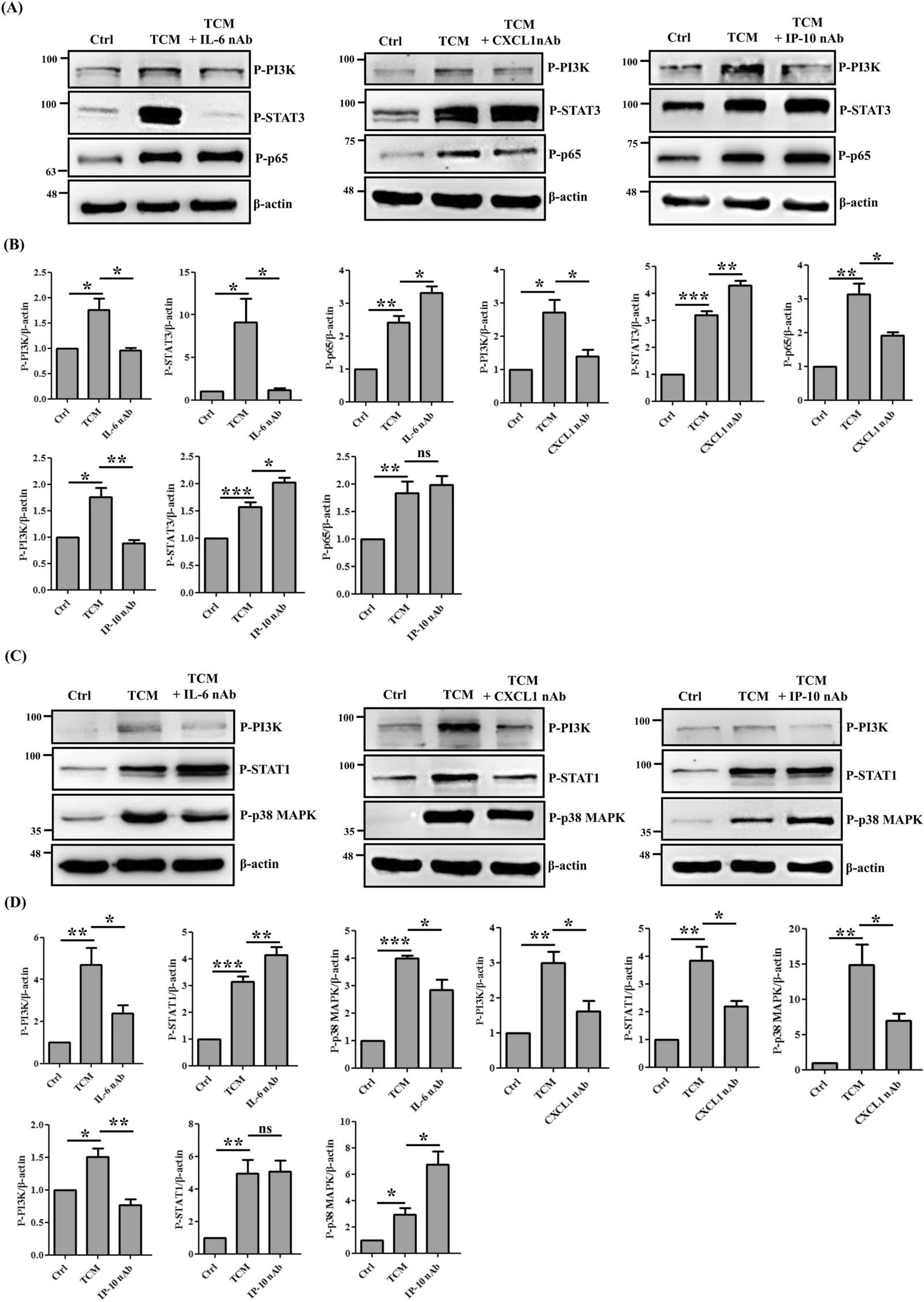
Neutralization of IL-6, IP-10, and CXCL1 in TCM differentially modulates TCM-induced signaling in THP-1 macrophages following short- and long-term exposure. **A** TCM was pretreated with neutralizing antibodies against IL-6, CXCL1, or IP-10, and co-cultured with THP-1 macrophages for 4 h. Whole cell lysates were collected, and the phosphorylation of PI3K, STAT3, and NF-κB p65 were analyzed by Western blot analysis and compared with those in TCM-treated and untreated control (Ctrl) groups. **B** Quantification of protein bands in (**A**) was performed using ImageJ software. **C** For long-term exposure, TCM was pretreated with the same neutralizing antibodies, and co-cultured with THP-1 macrophages for 24 h, and the phosphorylation of PI3K, STAT1, and p38 MAPK were analyzed as mentioned above. **D** Quantification of protein bands in **(C)**. *p < 0.05, **p < 0.01, and ***p < 0.001; *ns* Not significant

IL-6 deficiency has been shown to increase macrophage death and exacerbate lung inflammation, while exogenous IL-6 administration reduces inflammation and promotes macrophage survival [33]. Additionally, STAT1 and IP-10 have been implicated in promoting M1 macrophage polarization during extrapulmonary tuberculosis infection [34]. Given that TCM contains high levels of IL-6, CXCL1, and IP-10 secreted by Ect1 cells [27], we asked whether these cytokines contribute to the activation of specific signaling pathways in THP-1 macrophages upon short-term and long-term TCM stimulation. To address this, neutralizing antibodies against IL-6, CXCL1, or IP-10 were pre-incubated with TCM to block cytokine-mediated signaling prior to stimulation (Fig. 7A, B). We used ELISA to confirm effective neutralization of antibodies (1 µg/ml, 2 µg/ml, and 5 µg/ml) against their targets, and selected the lowest concentration (1 µg/ml) for subsequent experiments due to their sufficient neutralizing efficiency (Fig. S2). Notably, the phosphorylation of PI3K was suppressed in THP-1 macrophages upon short-term TCM stimulation for 4 h in the presence of IL-6, CXCL1, or IP-10 neutralizing antibody compared to TCM treatment alone. Additionally, STAT3 phosphorylation was attenuated by IL-6 neutralization but unexpectedly increased with CXCL1 or IP-10 neutralization. Moreover, NF-κB p65 phosphorylation was decreased upon CXCL1 neutralization but elevated with IL-6 neutralization, and there is no significant difference in NF-κB phosphorylation in response to IP-10 neutralization. Taken together, these results indicate that TV-induced IL-6 secretion from Ect1 cells positively regulates PI3K and STAT3 pathways while suppressing NF-κB activation in THP-1 macrophages upon short-term exposure to TV-induced inflammatory microenvironment. Additionally, TV-induced CXCL1 secretion promotes PI3K and NF-κB pathways while negatively regulating STAT3 signaling. Moreover, TV-induced IP-10 secretion upregulates the PI3K pathway while downregulating the STAT3 pathway.

Upon long-term stimulation with TCM for 24 h in the presence of IL-6, CXCL1, or IP-10 neutralizing antibody, phosphorylation of PI3K was markedly reduced in THP-1 macrophages (Fig. 7C, D). Additionally, STAT1 phosphorylation was decreased in response to TCM containing the CXCL1 neutralizing antibody but increased when IL-6 was neutralized. TV-induced IP-10 secretion appeared not to regulate the STAT1 pathway activation in macrophages. Furthermore, p38 MAPK phosphorylation was suppressed by IL-6 and CXCL1 neutralization, whereas IP-10 neutralization enhanced its activation. Consistent with the findings from short-term stimulation, long-term exposure to TV-induced IL-6, CXCL1, and IP-10 led to sustained activation of the PI3K pathway in macrophages. Additionally, prolonged stimulation with TV-induced IL-6 enhanced p38 MAPK activation while suppressing STAT1 signaling. In contrast, TV-induced CXCL-1 positively regulated all three TCM-enhanced pathways. Moreover, TV-induced IP-10 secretion appeared to suppress p38 MAPK signaling. Collectively, these results highlight the immunomodulatory roles of TCM-derived IL-6, CXCL1, and IP-10 in orchestrating macrophage signaling within the in vitro TV-induced inflammatory microenvironment.

### TCM promotes cervical cancer proliferation and migration but THP-1 macrophages counteract these effects in the TV-induced microenvironment

**Fig. 8:**
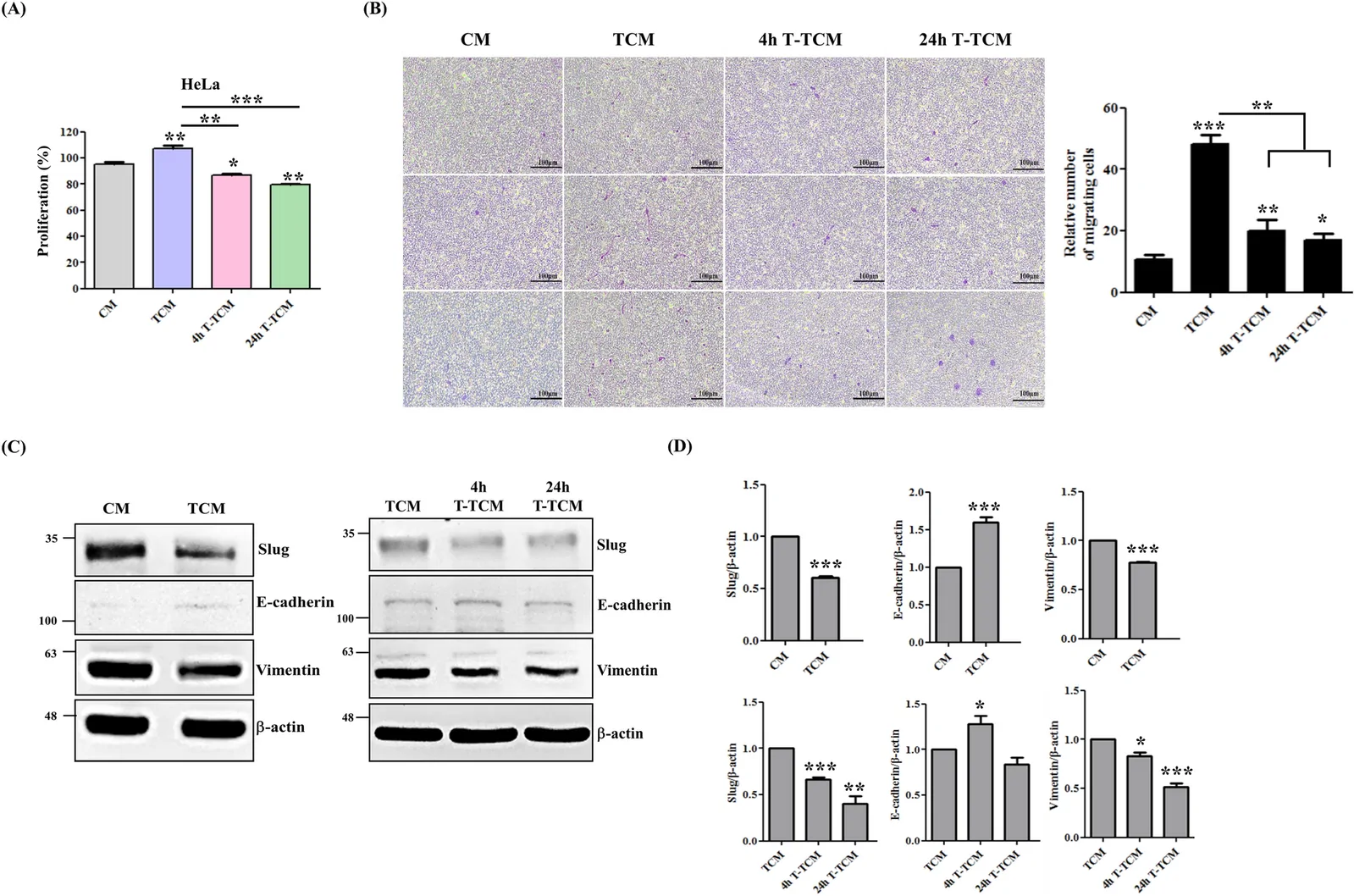
Effects of TCM and T-TCM treatment on HeLa cell proliferation, migration, and EMT-related marker expression. HeLa cells were treated with CM, TCM, or the conditioned medium from THP-1 macrophages co-cultured with TCM (T-TCM) for 4 h or 24 h. **A** Cell proliferation was assessed using the CCK-8 assay. **B** Cell migration was evaluated using a Transwell migration assay. **C** Expression levels of EMT-related proteins, including Slug, E-cadherin, and Vimentin, were determined by Western blot analysis using specific antibodies. β-actin served as an internal control. **D** Quantification of protein bands in **(C)** was performed using ImageJ software. Scale bar: 10 µm. *p < 0.05, **p < 0.01, ***p < 0.001

Our results demonstrate that TCM stimulation enhances the secretion of G-CSF, IP-10, and SCGF-β in THP-1 macrophages. Previous studies have shown that these cytokines are implicated in the progression of various cancers [35–37]. Similarly, conditioned medium derived from prostate cancer (PCa) cells stimulated with TV has been shown to enhance the production of CCL2, CXCL1, and IL-6, thereby promoting the proliferation, migration, and invasiveness of PCa cells [21]. These findings prompted us to investigate whether the TV-induced inflammatory microenvironment, along with THP-1 macrophages, contributes to the regulation of cervical cancer cell progression. To address this, TCM and conditioned medium collected from THP-1 macrophages following short- and long-term stimulation with TCM (4 h T-TCM and 24 h T-TCM, respectively) were collected and assessed for their effects on proliferation of HeLa cells. Notably, HeLa cells treated with TCM significantly increased proliferation compared to the CM-treated group (Fig. 8A). In contrast, exposure to T-TCM markedly reduced cell proliferation at both 4 h and 24 h compared with the TCM-treated group, with the reduction being more pronounced at 24 h. These results suggest that while the in vitro TV-induced inflammatory milieu promotes HeLa cell proliferation, the presence of macrophages within this microenvironment counteracts this effect. Additionally, HeLa cells treated with TCM exhibited enhanced migration compared to the CM-treated group. However, treatment with T-TCM significantly attenuated HeLa cell migration at both 4 h and 24 h compared with the TCM-treated group, with the greatest reduction observed following 24 h of treatment (Fig. 8B). These results indicate that while the TV-induced inflammatory microenvironment promotes cervical cancer cell proliferation and migration, macrophage-secreted factors within this microenvironment can suppress these tumor-promoting effects.

EMT is a critical process in cancer progression and metastasis, contributing to enhanced migration and invasiveness [38–41]. This process is typically characterized by the downregulation of E-cadherin and the upregulation of mesenchymal markers such as vimentin. The expression level of Slug, a transcription factor of the Snail family, has also been identified as a major initiator of the EMT process. We then assessed the effects of TCM and T-TCM treatments on EMT progression by evaluating the expression of EMT-related markers in HeLa cells. Unexpectedly, TCM treatment led to a significant upregulation of E-cadherin and a marked downregulation of Slug and vimentin relative to the CM-treated group (Fig. 8C, D). This result suggests that the EMT marker expression profile did not correlate with the observed increase in migration in HeLa cells in response to TCM treatment. Additionally, T-TCM treatment further enhanced E-cadherin expression and reduced Slug and vimentin levels in HeLa cells compared to the TCM-treated group (Fig. 8C, D). Collectively, these findings indicate that the microenvironment induced by TV infection promotes HeLa cell proliferation and migration. Moreover, THP-1 macrophages within the in vitro TV-induced inflammatory milieu attenuate proliferation and migration in HeLa cells.

## Discussion

Macrophages play a central role in the innate immune response by recognizing host- and pathogen-derived components, which trigger surface receptor modulation, phagocytosis, signaling transduction, and gene expression. Upon exposure to inflammatory stimuli, macrophages are able to release a wide range of cytokines [42]. A previous study indicated that conditioned medium from prostate epithelial cells stimulated with TV induced the production of the anti-inflammatory cytokines, including IL-10 and TGF-β, in macrophages [19]. In this study, we demonstrate that TCM stimulation promotes the secretion of several pro-inflammatory cytokines in macrophages. These findings suggest that the different cytokine profiles secreted from macrophages in response to the in vitro TV-induced inflammatory milieu may be attributed to the distinct epithelial cell types infected by TV. We found that short- and long-term exposure to TCM enhances the secretion of distinct cytokines in THP-1 macrophages, including SCGF-β, G-CSF, IL-6, IP-10, and IL-1ra. SCGF-β is a novel human growth factor that promotes the growth of primitive hematopoietic progenitor cells. Elevated levels of SCGF-β have been reported in lung and breast cancers [43, 44], and it has also been identified as a prognostic and predictive biomarker for chemotherapy response in advanced-stage hepatocellular carcinoma [20]. G-CSF has been shown to promote survival, proliferation, and differentiation of neutrophil precursors. Additionally, G-CSF can promote macrophage polarization toward the M2 phenotype that suppresses immune responses, including those against tumor cells [45, 46]. Hence, the enhanced SCGF-β and G-CSF secretion in macrophages within the TV-induced inflammatory milieu suggests their potential involvement in the establishment of an immunosuppressive and tumor-promoting microenvironment. Moreover, IL-6 is a pro-inflammatory cytokine and excessive production of IL-6 has been shown to lead autoimmune diseases and cancer development [47]. Hence, TCM-stimulated IL-6 secretion from macrophages may establish a more robust inflammatory microenvironment. IP-10 has been shown to be associated with the activation of pro-inflammatory M1 macrophages and able to kill tumor cells, inhibit angiogenesis, and promote adaptive immune responses [30, 48]. We found that TCM stimulation led to a decrease in IP-10 secretion in macrophages after short-term stimulation, suggesting that IP-10 may be utilized by macrophages during the early stage of TV infection. However, long-term stimulation with TCM results in marked secretion of IP-10. Previous studies have demonstrated that IP-10 is associated with HIV infection and higher viral loads during the early stages of HIV-1 infection in T cells [49, 50]. Hence, TCM-enhanced IP-10 production in macrophages may link to HIV transmission. IL-1 exerts pro-inflammatory effects by binding to the widely expressed IL-1 receptor type I (IL-1R1) [51]. IL-1ra can competitively bind to IL-1R1, thereby blocking IL-1-mediated activation of intracellular signaling [52]. Hence, long-term TCM-stimulated IL-1ra secretion may help suppress excessive IL-1-mediated inflammation. Collectively, our findings demonstrate that cytokines produced by macrophages in response to the in vitro TV-induced inflammatory milieu have multifunctional roles, contributing to both pro- and anti-inflammatory processes, and potentially influencing tumor progression or suppression.

IL-6 is associated with cancer progression, promoting tumorigenesis through activation of the JAK2/STAT3 signaling pathway [19, 53]. In our study, we also observed that short-term stimulation of macrophages with TCM induced STAT3 activation. Previous studies have shown that the gene expression of IP-10 is regulated by the MAPK and NF-κB pathways in microglial cells [31]. Additionally, G-CSF has been reported to activate the of ERK, PI3K/AKT, and NF-κB pathways in human trophoblast cells [54]. In murine macrophages, TV infection induces the production of IL-6, TNF-α, and IFN-γ via the MAPK and NF-κB pathways [14], and in SiHa cells, it activates the AKT, ERK, p38 MAPK, and JNK1/2 pathways [55]. These findings prompted us to further investigate the signaling pathways involved in TCM-induced cytokine secretion in macrophages. Our results demonstrate that short-term stimulation with TCM enhances the activation of the PI3K, NF-κB, and STAT3 pathways, whereas long-term stimulation activates the PI3K, p38 MAPK, and STAT1 pathways in THP-1 macrophages. These findings suggest that the immune response in macrophages upon stimulation with the in vitro TV-induced inflammatory milieu for different duration is dynamic. It is noteworthy that not all TCM-activated pathways positively regulate the secretion of TCM-induced cytokines in macrophages. For example, after long-term TCM stimulation, activation of the PI3K pathway inhibits IL-1ra secretion. While downregulation of IL-1ra via PI3K signaling may potentiate IL-1-mediated inflammatory signaling, its upregulation through TCM-enhanced p38 MAPK and STAT1 pathways may counteract this effect and protect cells from excessive inflammation. Additionally, both p38 MAPK and STAT1 pathways appear to negatively regulate G-CSF secretion, whereas PI3K signaling does not significantly influence G-CSF levels upon long-term stimulation. These findings suggest that TCM-enhanced G-CSF secretion upon long-term exposure to the in vitro TV-induced inflammatory milieu may be mediated by other uncharacterized pathways.

We further verified the roles of TCM-containing IL-6, CXCL-1, and IP-10 in regulating immune signaling in macrophages. Our findings demonstrated that neutralizing IL-6, CXCL1, and IP-10 in TCM by monoclonal antibodies leads to downregulation of PI3K signaling in macrophages following short- and long-term stimulation with TCM, indicating that these TV-enhanced cytokine secretions positively regulate the PI3K-mediated signaling cascades during early and late stages of TV infection. Given that PI3K activation is involved in macrophage polarization, cytokine production, and inflammatory responses [56, 57], our findings prove that TCM-secreted IL-6, CXCL1, and IP-10 consistently activate PI3K signaling, thereby contributing to diverse biological functions of macrophages during TV infection.

We observed that TV-induced IL-6 secretion within the microenvironment robustly enhances STAT3 phosphorylation in THP-1 macrophages during the early stages of infection, which is consistent with the established role of IL-6 in promoting inflammatory responses through the STAT3 signaling pathway in human vascular endothelial cells [58]. Although the overall STAT3 signaling was activated and this led to enhanced IP-10 secretion in THP-1 microphages, we found that TCM-enriched IP-10 negatively regulate STAT3 signaling. This finding may parallel the regulatory mechanism wherein STAT3 activation induces the expression of suppressor of cytokine signaling 3 (SOCS3), which in turn inhibits further STAT3 phosphorylation, forming a negative feedback loop to prevent excessive inflammatory responses [59, 60]. Additionally, IL-6 stimulation has been shown to activate both STAT1 and STAT3 signaling pathways [61, 62], though the extent and direction of this activation vary with cellular context. For example, activated T cells reduce IL-6-mediated STAT1 phosphorylation while maintaining STAT3 activation, as compared to resting T cells [63]. This selective inhibition of STAT1 has been suggested to limit pro-apoptotic and pro-inflammatory responses, while STAT3 activation supports cell survival [64, 65]. Consistent with these findings, our data showed that TCM-enriched IL-6 activates STAT3 signaling while suppressing STAT1 signaling at the early and late stages of TV infection, respectively. This coordinated regulation may attenuate STAT1-dependent pro-apoptotic signaling while promoting STAT3-mediated survival pathways in macrophages. However, the precise role of TCM-containing IL-6 in regulating macrophage survival remains to be elucidated. Additionally, IL-6 is known to activate NF-κB singling in intestinal epithelial cells [67]; however, we found that IL-6 neutralization led to enhanced NF-κB activation upon short-term exposure to TCM, suggesting that IL-6 in the TV-induced microenvironment may suppress NF-κB-mediated immune responses in THP-1macrophages at the early stage of TV infection.

CXCL1, also known as the growth-related oncogene, can recruit immune cells, promote inflammation, and induce angiogenesis [66]. In triple-negative breast cancer, CXCL1 activates several key signaling pathways, including PI3K, AKT, and NF-κB [67]. Consistent with these reports, we observed that CXCL1 in TCM positively regulated PI3K and NF-κB signaling in macrophages. In our previous study, we demonstrated that TV-EV treatment activates NF-κB signaling in both THP-1 macrophages and Ect1 cells, orchestrating host immune response during TV infection [18]. These findings suggest that CXCL1 in TCM may serve as a key driver of NF-κB activation in macrophages within the TV-induced microenvironment. Additionally, IP-10 has been reported to modulate the PI3K, p38 MAPK, and ERK pathways, or alternatively, to be modulated by these pathways [30, 68, 69]. Our results showed that TCM-containing IP-10 inhibited the activation of p38 MAPK signaling in macrophages. Similar to the role of IP-10 in negatively regulating STAT3 signaling during the early stage of TV infection, the inhibition of p38 MAPK by TCM-containing IP-10 can attenuate p38 MAPK-mediated IP-10 production in macrophages, leading to a negative feedback loop to control p38 MAPK signaling. Collectively, these findings demonstrate the distinct and context-dependent roles of TCM-containing CXCL-1 and IP-10 in modulating key signaling pathways such as PI3K, NF-κB, STAT3, and p38 MAPK, ultimately shaping immune responses in macrophages during TV infection.

Previous studies have reported that IL-6, CXCL1, and IL-8 can promote the proliferation and migration of cervical cancer cells [70–72]. Consistently, in our study, we observed that TCM significantly increased cervical cancer cell proliferation and migration compared to CM. Further investigations are needed to verify whether TCM-containing IL-6, CXCL1, and IP-10 exert cancer-promoting effects during TV infection. Unexpectedly, we found that the expression patterns of EMT-related proteins did not correlate with the enhanced migration of HeLa cells following TCM stimulation compared to CM. A previous study has reported that pre-EMT rat prostate cancer and human mammary epithelial cells exhibit greater migratory potential than their post-EMT counterparts [73], suggesting that enhanced migration is not necessarily a phenotypic feature of EMT. Additionally, in a pancreatic ductal adenocarcinoma mouse model, most tumors lose their epithelial phenotype through an alternative program involving protein internalization rather than transcriptional repression of epithelial genes, resulting in a partial EMT phenotype [74]. These findings suggest that cancer cell migration is not always positively correlated with EMT status and that cancer cells may undergo EMT through non-canonical programs. We further observed that co-culturing HeLa cells with T-TCM resulted in a greater reduction in cell proliferation and migration compared to TCM treatment alone. These findings imply that macrophages contribute to the formation of an anti-tumor microenvironment in the context of TV infection. However, the molecular mechanisms underlying these inhibitory effects remain to be elucidated. In particular, the potential contributions of T-TCM-enriched cytokines, including SCGF-β, G-CSF, IL-1ra, IP-10, and IL-6, to the observed suppression of HeLa cell proliferation and migration warrant further investigation. Beyond cytokines, other macrophage-derived components may also contribute to the regulation of these effects, such as THP-1-dervied extracellular vesicles and secreted proteins present in T-TCM. These factors represent promising candidates for future mechanistic studies.

We acknowledge several limitations in our current experimental design. First, while the conditioned medium derived from TV-stimulated Ect1 shares key cytokine profiles reported in clinical samples [75], it represents a simplified in vitro model that does not fully capture the polymicrobial environment or the dynamic, bidirectional interactions among epithelial cells, immune cells, and TV that occur during natural infection. Second, the use of immortalized cell lines, including THP-1, Ect1, and HeLa cells, limits the physiological relevance of our findings. Future studies employing primary human cervical epithelial cells and peripheral blood mononuclear cell (PBMC)-derived macrophages will be essential to validate our observations and enhance the physiological relevance and translational significance of this study. Notably, the Ect1 and HeLa cell lines utilized in this study are HPV positive. Although these HPV-containing cell lines provide consistent baselines under different experimental conditions, HPV oncoproteins (E6/E7) may modulate host immune signaling and cross-talk with TV-induced pathways. Additionally, the use of a single cervical cancer cell line (HeLa) may not adequately capture the biological heterogeneity of cervical cancer, thereby limiting the generalizability of our findings. Future studies incorporating additional HPV-positive cervical cancer cell lines (e.g., SiHa and CaSki) as well as an HPV-negative cell line (C33A) will be important to validate and strengthen our conclusions.

## Conclusions

**Fig. 9:**
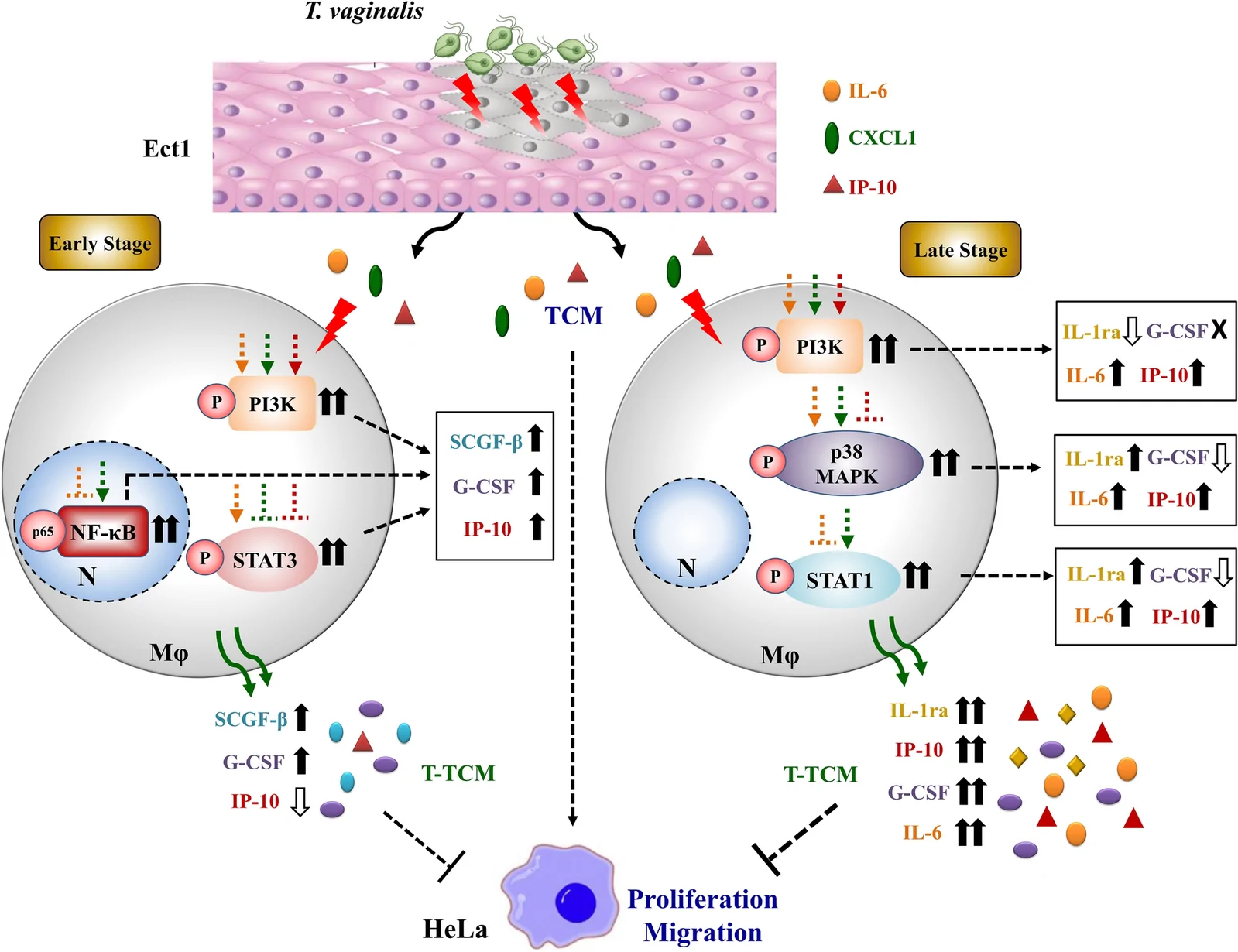
Proposed model for macrophage immune signaling and HeLa cell behavioral changes driven by the in vitro TV-induced inflammatory milieu. In the TV-shaped microenvironment, TV-induced IL-6, IP-10, and CXCL1 secretion from Ect1 cells modulates the PI3K, NF-κB, and STAT3 signaling pathways in THP-1 macrophages during the early stage of infection, while modulating PI3K, p38 MAPK, and STAT1 signaling pathways during the late stage of infection. These signaling events lead to the release of various cytokines, which may contribute to the suppression of proliferation, migration, and EMT process in cervical cancer HeLa cells. Dashed lines indicate proposed mechanisms based on the findings of the present study

Collectively, our results demonstrate that the inflammatory microenvironment generated by TV-stimulated Ect1 cells enhances the secretion of specific cytokines in THP-1 macrophages, potentially regulating pro-inflammatory and anti-inflammatory responses, as well as tumor development during TV infection (Fig. 9). Additionally, the in vitro TV-induced inflammatory milieu differentially modulates the activation of signaling pathways in THP-1 macrophages through the secretion of IL-6, CXCL1, and IP-10, and enhances HeLa cell proliferation and migration. It remains to be determined whether these TCM-enriched cytokines directly modulate the proliferation and migration of HeLa cells. Furthermore, our findings suggest that TCM-enhanced migration may be mediated through an EMT-independent mechanism. Conversely, the microenvironment established by co-culturing THP-1 macrophages with TCM (T-TCM) led to decreased proliferation, migration, and EMT progression in HeLa cells. These findings highlight the complex immunomodulatory roles of TV-induced cytokines in driving macrophage responses within the vaginal microenvironment. Additionally, this study provides new insights into how the TV-induced microenvironment, with or without the presence of macrophages, influences cervical cancer cell behavior, thereby advancing our understanding of the dynamic interplay among TV, macrophages, and cervical cancer cells during infection.

## Supplementary Information

Supplementary Material 1. Fig. S1 Viability of THP-1 macrophages following TCM or CM treatment. THP-1 macrophages were exposed to TCM or CM for 24, 48, or 72 h, and cell viability was assessed using the CCK-8 assay.

Supplementary Material 2. Fig. S2 Neutralizing efficiency of antibodies against IL-6, CXCL-1, and IP-10. TCM was pretreated with neutralizing antibodies targeting IL-6, CXCL-1, and IP-10 at concentrations of 1, 2, or 5 µg/ml for 1 h, and the residual cytokine levels were quantified by ELISA

## Data Availability

All data generated or analyzed during this study are included in this published article.

## Abbreviations

TV: *Trichomonas vaginalis*
STIs: Sexually transmitted infections
Ect1: Ectocervical cells
HIV: Human immunodeficiency virus
TLR2: Toll-like receptor-2
IL-6: Interleukin-6
IFN-γ: Interferon-gamma
TNF-α: Tumor necrosis factor-alpha
NF-κB: Nuclear factor kappa-light-chain-enhancer of activated B cells
MAPK: Mitogen-activated protein kinase
TGF-β1: Transforming growth factor-β
STAT1: Signal transducer and activator of transcription 1
CM: Condition medium of Ect1 cells without TV exposure
TCM: Conditioned medium of Ect1 cells stimulated with TV
T-TCM: Conditioned medium of THP-1 macrophages stimulated with TCM
KSFM: Keratinocyte serum-free medium
EGF: Epidermal growth factor
PI3K: Phosphoinositide 3-kinase
ERK: Extracellular signal-regulated kinase
SCGF-β: Stem cell growth factor-β
G-CSF: Granulocyte colony-stimulating factor
EMT: Epithelial–mesenchymal transition
PCa: Prostate cancer
IL-1R1: IL-1 receptor type I
SOCS3: Suppressor of cytokine signaling 3
